# Sarcoidosis Epidemiology: Race Matters

**DOI:** 10.3389/fimmu.2020.537382

**Published:** 2020-09-15

**Authors:** Kerry Maryse Hena

**Affiliations:** Division of Pulmonary, Critical Care, and Sleep Medicine, Department of Medicine, New York University, New York, NY, United States

**Keywords:** sarcoidosis, black race/ethnicity, sarcoidosis epidemiology, sarcoidosis mortality, implicit bias, health equity

## Abstract

Rather than a single disease entity, sarcoidosis may be a constellation of “sarcoidoses” with a characteristic pattern of organ involvement and clinic course, depending upon the triggering exposure and underlying epidemiologic factors such as race. This review examines the racial disparities inherent to sarcoidosis disease course and mortality and discusses factors that may be responsible for these findings. In the United States, black patients with sarcoidosis experience more severe pulmonary disease, more multiorgan involvement, and an overall worse prognosis with higher rates of hospitalization and mortality. Beyond inherent genotype, ascertainment and access to medical care, physician implicit bias, and patient perceived discrimination likely play a role. Moving forward, epidemiologic concepts can be used to formulate strategies for control, treatment, and even prevention of disease in black Americans at risk for developing life-altering or life-threatening sarcoidosis phenotypes. Identification and rectification of modifiable risk factors such as socioeconomic status, lack of insurance, and financial barriers to care as well as the incorporation of implicit bias training for physician will likely lead to improvement in discordant outcomes.

## Introduction

Sarcoidosis is a multisystem disease characterized by granulomatous inflammation. Although its etiology remains unknown, sarcoidosis is believed to represent a genetically primed abnormal immune response to an antigenic exposure (1). Rather than a single disease entity, sarcoidosis may be a constellation of “sarcoidoses” with a characteristic phenotype, depending upon the triggering exposure and underlying genotype. As such, race plays an important role in sarcoidosis epidemiology, disease presentation, and clinical outcomes (2).

In the United States, in particular, different phenotypic expressions are found between black and white subjects, likely resulting from genetics, environment, socioeconomics, implicit or racial biases, or a combination thereof. Given the current political climate and impetus for change in the United States, it is important to shed light on discordant clinical outcomes in vulnerable patient populations. This article examines the racial disparities inherent to sarcoidosis disease course and mortality and discusses factors that may be responsible for these findings.

## Incidence and Prevalence

Consistently, black subjects are reported to be most affected by sarcoidosis, black females even more so. Studies reporting incidence and prevalence of sarcoidosis, broken down by race and sex if available, are presented in Table 1.

**Table 1:** Sarcoidosis incidence estimates reported in the literature.

**References**	**Country**	**Sex, race/ethnicity**	**Time period**	**Incidence per 100,000**	**Data source**
Arkema et al. (3)	Sweden	Male and female, race not reported	2003–2013	11.5	National Patient Register
Baughman et al. (4)	USA	Male and female, multiracial	2010–2013	Black: 17.8 White: 8.1 Hispanic: 4.3 Asian: 3.2	Optum Health Care Database
Coquart et al. (5)	Guadeloupe, West Indies	Male and female, Afro Caribbean	2003–2010	2.3	Hospital and laboratory databases
Cozier et al. (6)	USA	Female, black	1995–2007	71	Black Women's Health Study, self-reported sarcoidosis
Duchemann et al. (7)	France, Greater Paris	Male and female, multiracial	2012	Overall: 4.9 Afro Caribbean: 16.9 North African: 9.7 Other: 6.4 European: 2.4	Hospitals in Seine-Saint-Denise County; social security
Dumas et al. (8)	USA	Female, multiracial	1989–2011	Overall: 11 Black: 43 White: 11	Nurses' Health Study II, self-reported
Hillerdal et al. (9)	Sweden, Uppsala County	Male and female, race not reported	1966–1980	Overall: 19 Male: 16.5 Female: 21.7	General Health Screening
Rybicki et al. (10)	USA, Detroit, MI	Male and female, multiracial	1990–1994	Black: 35.5 White: 10.9	Health Alliance Plan HMO
Ungprasert et al. (11)	USA, Olmsted County, MN	Male and female, 90% white	1976–2013	Male: 10.5 Female: 11	Rochester Epidemiology Project

Dating back to the 1960's, researcher Andrew Z. Keller reported significantly different frequency distributions of sarcoidosis with black patients being more commonly diagnosed and treated than whites (12). In the 1990's, an analysis of race in patients from Detroit, Michigan suggested that the relative rates of sarcoidosis in black compared to white patients was as high as 4:1 (10.9/100,000 per year in whites and 35.5/100,000 in blacks) (10). In this study by Rybicki and colleagues, the highest annual age-specific incidence was found in black females aged 30–39 years (107/100,000) (10). A later analysis of sarcoidosis based on U.S. managed healthcare use from 2009 to 2013 showed that the incidence and prevalence of sarcoidosis was significantly higher for black (17.8 and 141.4 per 100,000) than for white individuals (8.1 and 49.8); the highest prevalence for sarcoidosis was again noted in black females (178.5) (4). Of note, additional minority populations, such as Hispanic and Asian patients, were significantly less likely to have sarcoidosis than black or white patients, consistent with what has been suggested in the past (4, 13). More recently, amongst participants in the Black Women's Health Study, incidence rates were observed as high as 71 per 100,000 per year in African American females (6).

In summary, sarcoidosis incidence and prevalence vary greatly based on race with a strong predilection for black populations in the United States, which may be the result of race-specific genetic associations and environmental socioeconomic factors.

## Genetic Predisposition

As stated previously, sarcoidosis likely results from an environmental insult in a genetically susceptible host. The high incidence and prevalence of sarcoidosis in black subjects suggests an inherent genetic susceptibility. In fact, first-degree relatives of black sarcoidosis cases have about a 3-fold disease risk increase (14).

Multiple sarcoidosis susceptibility loci have been explored in candidate gene and genome-wide association studies. These include the highly polymorphic human leukocyte antigen (HLA) genes, residing in the class II major histocompatibility complex (MHC) on chromosome 6, as well as other genes that influence antigen processing and presentation, T-cell activation and recruitment, and granulomatous inflammation at the core of sarcoidosis immunopathogenesis. A particular HLA allele, DQB1^*^0602, was shown to confer both increased susceptibility to sarcoidosis and a predilection for radiographic progression of disease in a family-based genetic association analysis of sarcoidosis in U.S. black families (15). Conversely, Butyrophilin-like 2 (BTNL2), which also resides in the class II MHC region on chromosome 6 and probably functions as a T-cell co-stimulatory molecule, has been shown to have a more modest association with sarcoidosis in black compared to white populations; this may be due to greater allelic diversity or an antagonistic effect of HLA class II risk alleles on BTNL2-associated risk in black samples (16). Amongst black siblings enrolled into the Sarcoidosis Genetic Analysis Consortium (SAGA), a genome scan found the strongest linkage signal on chromosome 5 (17). Subsequent fine mapping studies indicated a sarcoidosis susceptibility gene on chromosome 5q11.2 and a gene protective effect on 5p15.2 (18). Further stratification of black families by genetically determined ancestry revealed linkage differences by subpopulation, with previously reported linkage signals at 1p22, 3p21-14, 11p15, and 17q21 specific to ancestral heritage (19).

In all, given the identification of multiple candidate genes and suggestive regions for linkage, it is likely that more than one gene influences sarcoidosis susceptibility and disease presentation in black populations. This is further confounded by ancestral differences in admixed black communities residing in the United States.

## Disease Presentation

In sarcoidosis the granulomatous inflammation can affect virtually all organs and tissues. However, disease presentation, including extent and severity of disease, has been shown to vary based on demographic variables such as race. A Case-Control Etiologic Study of Sarcoidosis (ACCESS)—a prospective, multicenter study of sarcoidosis patients enrolled within 6 months of diagnosis—provided an opportunity to examine the association of demographic measures, socioeconomic status and barriers to care with severity of disease at presentation, as defined by extent of organ involvement, chest radiographic staging and basic spirometry (20, 21).

As far as organ involvement at presentation, ACCESS showed that black patients tend to experience more extrapulmonary sarcoidosis, affecting the skin (other than erythema nodosum), bone marrow, liver, extrathoracic lymph nodes and eyes (20). Calcium dysmetabolism, on the other hand, was more common in white patients (20). Only pulmonary involvement was independent of age, sex and race (20). Later ACCESS analyses demonstrated that lower income, the absence of private or Medicare health insurance, and other barriers to care were associated with sarcoidosis severity at presentation, as were black race and female sex (21). In comparison to ACCESS whites, ACCESS blacks were more likely to have more organs involved and lower FVC % predicted as well as family income of <US$20,000 and to have other public insurance such as Medicaid (21).

Therefore, in addition to a suspected genetic predisposition for more severe pulmonary and extrapulmonary disease, the association of black race with socioeconomic status (low income, public insurance) and severity of disease at presentation suggests that black patients are more likely to have financial barriers to care and resultant delay in seeking care than their white counterparts (21). Additionally, implicit bias—any unconsciously-held set of associations about a social group—impacts the behavior of both physicians and black patients, with physicians potentially discounting symptoms of sarcoidosis in black patients until more severe and/or black patients delaying care out of medical mistrust or perceived discrimination (22–25).

## Clinical Course

While sarcoidosis tends to improve or remain stable in the majority of patients, longitudinal studies have shown worse outcomes are associated with black race and/or lower annual family income (26, 27). In the ACCESS 2 years follow-up study, black patients had a higher likelihood of decreasing FVC and developing new organ involvement over time (26). Similarly, clinical data from a large cohort of sarcoidosis patients followed at Medical University of South Carolina over a 12-years period, showed that black patients had more advanced radiographic stages of sarcoidosis, more organ involvement, and more frequently required anti-sarcoidosis medication compared to white patients (27).

Furthermore, black patients tend to have a higher rate of hospitalization for sarcoidosis. An analysis of the National Hospital Discharge Survey (NHDS) from 1979 to 2000 showed that mean rates of hospitalization were nine time higher for black patients (28). A retrospective study of the Nationwide Inpatient Sample supported this trend, demonstrating a near doubling of hospitalizations among sarcoidosis patients, with disproportionate rate increases in black, female, and older patients from 1998 to 2008 (29).

As such, black patients with sarcoidosis experience worse prognosis, more multiorgan involvement, and a higher rate of hospitalization. Beyond inherent genotype, ascertainment and access to preventative and primary care may also be playing a role in this disparity of outcomes for black individuals, as is implicit bias.

## Mortality

Many patients with sarcoidosis have a benign clinical course, but for some, it is a chronic, life-altering, and even fatal disease. While overall mortality rates from sarcoidosis have been shown to be increasing, significant differences exist based on race. Data from the National Center for Health Statistics (NCHS), demonstrated a 3% average yearly increase in mortality rate for all-comers with sarcoidosis from 1988 to 2007 (30). However, the greatest absolute increase in sarcoidosis-related deaths was among black females (10 deaths per million) followed by black males (three deaths per million), in comparison to at most one death per million in white males or females (30). The most common cause of death was sarcoidosis itself; younger sarcoidosis decedents with pulmonary fibrosis were more likely to be black than white and have a cardiac involvement contribute to death. A later study using NCHS data from 1999 to 2010 with a focus on racial and sex disparities, showed a 12 times higher age-adjusted mortality rate for black patients compared with white patients (16 vs. 1.3 per million, respectively) (31). Again, black sarcoidosis decedents died at an earlier age and more often had associated pulmonary hypertension (31). An early study by Gideon and Mannino reported a U.S. sarcoidosis mortality ratio, black: white, of 14:1 (32).

This race-specific trend of higher mortality from sarcoidosis at a younger age in black patients has multiple potential explanations, some more alarming than others. Hypotheses include increased incidence and severity of disease, a predilection for the lethal complications of pulmonary fibrosis and pulmonary hypertension, as well as long-standing systemic health and social inequities.

## Racism

As the Covid-19 pandemic has so powerfully illustrated, race plays a significant role as a determinant of health in the United States (33). Beyond socioeconomic factors, perceived discrimination has been linked to adverse health outcomes in mental and physical health domains (23–25). In addition to implicit or racial biases, the perception of discrimination or stigma of inferiority appears to induce physiological and psychological arousal with direct deleterious health consequences (34). While this has not been elucidated specifically in sarcoidosis, the association of perceived discrimination with poorer health outcomes has been explored across a broad range of outcomes, including cardiac disease, renal insufficiency and subclinical cerebrovascular disease (23–25).

## Future Directions

Despite its nineteenth century origin and more than 100 years of inquiry, much about sarcoidosis is still unknown. Perhaps this is because of its many points of divergence—its variant epidemiology, varied triggering exposures and ultimately variable clinical phenotypes or “sarcoidoses.” Regardless, its higher contraction and death rates in black Americans deserves further attention.

Ideally, future studies performed will improve our understating of the relationships among exposure, race and other epidemiologic factors, with current clinical phenotypes and outcomes. Such endeavors should include large, matched epidemiologic studies conducted in racially diverse populations, facilitated by large population data sets and disease registries. In addition to facilitating etiologic hypotheses of the exposure-host interaction at the core of sarcoidosis pathophysiology, epidemiologic concepts should be used to formulate strategies for control, treatment, and even prevention of disease in Black Americans who are at the greatest risk for developing life-altering or life-threatening disease. Identification and rectification of modifiable risk factors such as socioeconomic status, lack of insurance, and financial barriers to care will likely lead to improved outcomes. Making anti-racism or implicit bias training an essential professional competency would equip physicians with the tools needed to address racism and its adverse health effects (35).

Finally, these findings should influence research in the areas of health disparities and disease processes among various racial groups and between the sexes throughout the world. Achieving health equity requires valuing all individuals and populations equally, recognizing and rectifying historical injustices, and providing resources according to need. Health disparities will be eliminated *only* when health equity is achieved (36).

## Author Contributions

KH contributed a race-centered review of sarcoidosis epidemiology, highlighting racial disparities inherent to sarcoidosis disease course and mortality and discusses factors that may be responsible for these findings. She was the sole contributing author for this section.

## Conflict of Interest

The author declares that the research was conducted in the absence of any commercial or financial relationships that could be construed as a potential conflict of interest.
